# Outbreak of Sepsis Following Surgery: Utilizing 16S RNA Sequencing To Detect the Source of Infection

**DOI:** 10.7759/cureus.22487

**Published:** 2022-02-22

**Authors:** Eran Segal, Shahar Bar Yosef, Alex Axel, Naty Keller, Francisc Shlaeffer, Amnon Amir, Gilat Efroni, Yahel Haberman

**Affiliations:** 1 Anesthesiology, Intensive Care and Pain Medicine, Assuta Medical Center, Tel Aviv, ISR; 2 Infectious Diseases, Assuta Medical Center, Tel Aviv, ISR; 3 Department of Health Sciences, Ariel University, Ariel, ISR; 4 Gastroenterology Research Laboratory, Sheba Medical Center, Ramat Gan, ISR; 5 Pediatric Gastroenterology, Sheba Medical Center, Ramat Gan, ISR

**Keywords:** hospital-aquired infection, nosocomial infections, anesthesia complications, postoperative sepsis, citrobacter freundii, hospital infection control

## Abstract

Background

Nosocomial infections are a significant health concern. Following surgery, infections are most commonly associated with the surgical site, yet there are other potential sources for infections after surgical interventions. Identification of the source of infections can be very challenging.

Methodology

An outbreak of postoperative infections following surgery led to intensive care unit (ICU) admission of patients immediately after the surgical procedure. The blood cultures of two patients were positive for *Citrobacter freundii*. The only connection between all cases was the anesthesiologist. An epidemiological inquiry could not definitively identify the source of the outbreak. Therefore, we utilized an RNA sequencing technique to evaluate the microbiome of the anesthesiologist and compared the results to bacteria cultured from the bloodstream of the two patients.

Results

The anesthesiologist’s microbiome contained amplicons that were identical to those of the bacteria in the patient’s bloodstream. Because *Citrobacter freundii *is an uncommon source of bloodstream infections, and in the normal human microbiome, the results establish the source of a cluster of infections to the anesthesiologist.

Conclusions

In cases of nosocomial infections, when conventional microbiological techniques do not clearly establish the source of the infection, using 16S RNA sequencing should be considered.

## Introduction

Various sources cause outbreaks of infections following surgery. These include healthcare personnel, environmental sources, contaminated medications, or the patients themselves. Several studies have examined various operating room parameters, including staff attire [1], hand hygiene practices [2], prophylactic administration of antibiotics, method of cleaning the anesthesia workplace [3], and vascular catheter care [4].

When several infectious episodes occur over a short period of time, an investigation into the source of the infections should be performed, and an epidemiological effort to identify the cause of the outbreak should be made. If a clear source is found, actions can be taken to prevent further risk. In some cases, identifying the source of the infections using conventional microbiological tools may not be sufficient, necessitating the use of more advanced molecular techniques [5].

In this study, when conventional microbiological methods failed, we used 16S RNA sequencing to establish the source of infection during an outbreak following surgery. Using this technique, we found that the anesthesiologist who was involved in the care of all patients carried the organism causing the infection, *Citrobacter freundii*, in his microbiome. This enabled us to establish the source of the outbreak and provide further training to the clinician to improve compliance with infection prevention guidelines.

## Materials and methods

Description of the episode

A patient who underwent dilatation and curettage (D&C) on January 16, 2020, for a missed abortion, developed a clinical picture of sepsis including fever (temperature >38°C) and hypotension (mean arterial pressure <60 mmHg). She was admitted to the intensive care unit (ICU) and treated with antibiotics for suspected sepsis due to the surgical procedure. A few hours later, another patient who had also undergone a D&C was admitted to the ICU due to sepsis. The following day, we learned that two more women who underwent the same procedure had signs of an infectious episode and were being treated at two other hospitals for a clinical picture suggestive of sepsis.

On January 17th, a male patient who had a revision of total knee replacement surgery developed a septic clinical picture shortly after the surgery, was admitted to the ICU, and required intubation and mechanical ventilation due to acute respiratory distress syndrome. On the following day, two patients who had undergone colporrhaphy the day before complained of fever, weakness, and hypotension. They were both admitted to the ICU and diagnosed with sepsis based on their clinical presentation and laboratory results of white blood cell count, C-reactive protein level, and procalcitonin levels. Details of the patients are presented in Table 1.

**Table 1:** Clinical details of the patients. D&C: dilatation and curettage; TKR: total knee replacement; LMA: laryngeal mask airway

Patient	Date	OR	Procedure	Surgeon	Anesthesiologist	Anesthetic technique
1	16/1	A	D&C	B	A	General-Mask
2	16/1	A	D&C	B	A	General-Mask
3	16/1	A	D&C	B	A	General-Mask
4	16/1	A	D&C	B	A	General-Mask
5	17/1	B	TKR	C	A	Spinal and sedation
6	17/1	B	Colporrhaphy	D	A	General-LMA
7	17/1	B	Colporrhaphy	Ds	A	General-LMA

An epidemiological investigation was initiated to determine a possible connecting link between the cases. Staff members were questioned regarding personal medical issues, hand hygiene, procedures involving equipment, and medications. There was no change from usual practice.

The investigation revealed that the anesthesiologist in all cases was the same physician. No other clinician was involved in the care of all patients. When questioned, he denied any current illness or unusual exposure. He had anesthetized six women on the same day as the four patients with D&C before these cases. None of them had any complaints either immediately after the procedures or when questioned three days later by phone. The two operating rooms in which the cases occurred are located on different floors of the hospital. The sterile equipment used in the procedures was similar for the first four cases but different in the last three cases.

As part of the clinical assessment of the patients, the Sequential Organ Failure Assessment (SOFA) score was calculated. The maximal score of the patients was correlated with the duration of surgery. Statistical analysis was performed with JMP software (SAS Institute, Cary, NC, USA).

Blood cultures were obtained from the five patients who were treated in our hospital. Patients five and seven had positive cultures. Both cultures grew the same anaerobic Gram-negative organism: *Citrobacter freundii*. Patient seven also had an aerobic Gram-negative bacillus: *Acinetobacter baumannii*, cultured from the same blood specimen. Because the anesthesiologist was the only identified common factor involved in the care of all patients, we decided to test the possibility of him being the source of the infection. Cultures of the anesthesiologist’s mouth, nares, and hands were taken three times in the days following the events and were negative for Gram-positive or Gram-negative pathogenic bacteria.

We then obtained samples from different body sites to characterize his microbial composition using V4 16S rRNA amplicon metagenomic sequencing. Swabs were obtained from his nares, buccal area, hands, and feces (physician A), and from another anesthesiologist who served as control (physician B). We also sequenced using V4 16S rRNA amplicon metagenomic sequencing the three isolated bacteria that were grown and identified as *Citrobacter freundii* (two isolates from patients five and seven) and *Acinetobacter baumannii* from patient seven.

16S rRNA gene amplicon sequencing and analyses

DNA extraction and polymerase chain reaction (PCR) amplification of the variable region 4 (V4) of the 16S rRNA gene using Illumina adapted universal primers 515F/806R was conducted using the direct PCR protocol [Extract-N-Amp Plant PCR Kit (Sigma-Aldrich, Inc.)], as previously described [6]. Briefly, PCRs were conducted and amplicons were pooled in equimolar concentrations into a composite sample that was size selected (300-500 bp) using agarose gel to reduce non-specific products from host DNA. Procedural blanks were samples that included all buffers, reagents, and procedures but had no DNA template to account for potential procedural contaminants. Sequencing was performed on the Illumina MiSeq platform with the addition of 15% PhiX, generating paired-end reads of 175 bp in length in each direction.

Reads were processed in a data curation pipeline implemented in QIIME 2 version 2019.4 [7]. Reads were demultiplexed according to sample-specific barcodes. Quality control was performed by truncating reads after three consecutive Phred scores lower than 20. Reads with ambiguous base calls or shorter than 150 bp after quality truncation were discarded. Amplicon sequence variants (ASVs) detection was performed using Deblur [8]. Reads were then truncated to 150 bp. Taxonomic classification of ASVs was performed using blast, specifically examining ASVs that were identified by this approach in the isolated culture, including “TACGGAGGGTGCAAGCGTTAATCGGAATTACTGGGCGTAAAGCGCACGCAGGCGGTCTGTCAAGTCGGATGTGAAATCCCCGGGCTCAACCTGGGAACTGCATCCGAAACTGGCAGGCTAGAGTCTTGTAGAGGGGGGTAGAATTCCAGG” for *Citrobacter freundii*, and “TACAGAGGGTGCGAGCGTTAATCGGATTTACTGGGCGTAAAGCGTGCGTAGGCGGCTTTTTAAGTCGGATGTGAAATCCCCGAGCTTAACTTGGGAATTGCATTCGATACTGGGAAGCTAGAGTATGGGAGAGGATGGTAGAATTCCAGG” for *Acinetobacter baumannii*. A heatmap was generated using Calour version 2018.10.1 with default parameters [9].

We have uploaded the 16S files to NCBI SRA. The SRA accession is SPRJNA803025.

Approval for publication of this epidemiological event was provided by the Institutional Review Board of Assuta Medical Centers (approval number: 0056-20-ASMC).

## Results

Clinical description

The clinical information regarding the patients is presented in Table 1. The four patients treated on January 16, in OR A, had short procedures that lasted 10-15 minutes. They were all performed under general anesthesia with a face mask. The three patients who were operated on, on the 17th, underwent the surgery in OR B in the main OR suite of the hospital. The same anesthesiologist provided anesthesia. The first procedure was an orthopedic procedure performed under spinal anesthesia with sedation, which lasted four hours. The following two procedures were performed consecutively in the same room, the surgeon was a gynecologist, and anesthesia was delivered by the same anesthesiologist with general anesthesia using a laryngeal mask airway. Laboratory results are presented in Table 2. Data are available for the five patients who were treated in our hospital.

**Table 2:** Clinical data for the five patients who were treated in our ICU following the septic event. SOFA: Sequential Organ Failure Assessment; ICU: intensive care unit

Patient	Surgery duration (minutes)	Procalcitonin (ng/mL)	C-reactive protein (mg/L)	Highest system organ failure (SOFA) score
1	15	37	323	3
2	15	46	254	1
5	350	70	156	9
6	120	53	225	6
7	120	22	100	4

**Figure 1:**
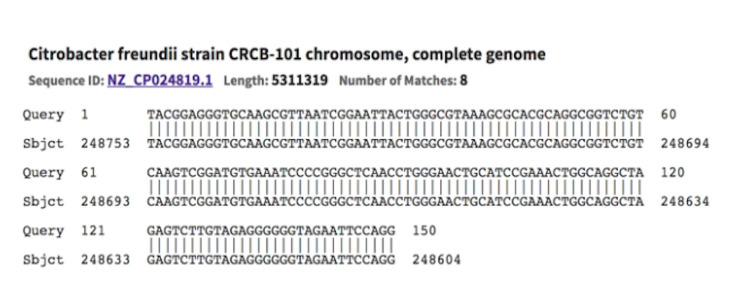
Blast result of the ASV from positive blood cultures with Citrobacter freundii and Anesthesiologist A samples (stool and hands). ASV: amplicon sequence variant

As shown in Figure 1, the ASVs from the isolated cultures identified as *Citrobacter freundii* were the same as the obtained 16S sequence in the NCBI blast.

**Figure 2:**
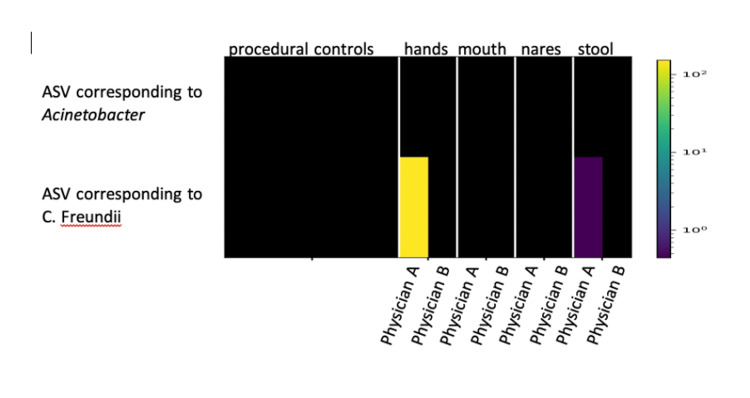
Heat map of the presence of ASVs of Citrobacter freundii. Presence of ASV in samples from different sites of physician A, physician B, and controls. The ASV of *Citrobacter freundii*, which were present in the blood cultures of patients five and seven, are only detected in the hands of physician A, and at a lower quantity in his stool. No ASV was detected in samples from physician B or in any of the controls. ASVs of *Acinetobacter baumanii* were not detected in any of the physician’s samples or controls. ASV: amplicon sequence variant

Moreover, this identical ASV was identified in physician A hands and fecal samples but not in physician B samples or in any of the negative procedural controls (Figure 2). This clearly indicates that it was not a procedural contaminant. In contrast, *Acinetobacter baumannii* ASV was identified only in the culture but not in physician A or B samples or in any of the procedural blank samples.

## Discussion

Nosocomial infections are a common occurrence, and the source of infection is frequently ascribed to bacterial transfer from patient to patient, most commonly by staff. Other possible sources are the patient’s flora, and, in some cases, the possibility of a clinician being the source of the infectious organism has also been reported.

The possible contribution of anesthetic practice to nosocomial infections has received less attention than other possible sources. Although perioperative nosocomial infections are a significant portion of hospital-acquired infections, these are most commonly due to surgical technique, staff involved directly with the surgical wound, patients themselves, or poorly sterilized equipment. There have been several reports of anesthetic medications, particularly propofol, as a source of infection, particularly if single patient vials of the medication are used in more than one patient [10-12]. An extreme case of an anesthesiologist infecting a group of patients with hepatitis over a long period by using the same syringe on himself as on the patients has also been described [13]. Transmission of hepatitis to a patient has also been described during a procedure that was not prone to transmission [14]. In 2000, Ross et al. described a case of an anesthesia assistant who incurred a wound on his finger, which led to infection of hepatitis from a patient, followed by his transmission of the virus to other patients. They noted that he did not adhere to hand hygiene guidelines and allowed the wound to be openly exposed while involved in patient care [15].

During routine anesthetic care, the most probable mechanism by which an anesthesiologist can cause infection in a patient is through contamination of the anesthesia workplace, particularly the anesthesia machine after airway management, and especially the areas which are frequently touched during anesthesia, namely, the adjustable pressure limit valve and the ventilatory settings controls [16,17]. The anesthesia cart, where most medication syringes are placed and returned to after the drug is administered, also becomes contaminated rapidly during anesthesia. Contaminated areas can then become a source of bacterial exposure, via the anesthesiologist’s hands, to the next patient. Use of appropriate hand hygiene, as well as correct donning and doffing of gloves, can decrease this; however, it has been reported that compliance of anesthesiologists with the required hand hygiene is very low [18]. This may be due to significant deficits in knowledge among anesthesia providers regarding correct hand hygiene practices [19]. In addition, awareness of possible transmission due to equipment used by anesthesiologists may be lacking. A study of awareness among healthcare clinicians regarding stethoscopes as a possible source of nosocomial infection reported that most believed that their hospitals did not have protocols for disinfecting stethoscopes and there was no accountability regarding this [20].

In many cases, it may not be feasible to comply with the guidelines because of the sheer number of hand hygiene opportunities in a fast-paced scenario such as an anesthetic induction [21]. For example, it is very difficult for an anesthesiologist who performed intubation to replace gloves and perform hand hygiene before touching the anesthesia machine to prevent transferring bacteria from the patient’s airway to the anesthesia machine.

An expert guidance protocol for the prevention of contamination of anesthesia workstations was recently published [22]. This guideline addresses many of the questions pertaining to appropriate techniques for infection prevention practiced by anesthesiologists. The recommendations for hand hygiene are to comply with the World Health Organization’s guidelines. This means applying hand hygiene on entry and exit from the OR, before insertion of an intravascular catheter, before drawing up medications from ampules or vials, and every time the hands are soiled such as after airway management, or contact with the patient’s secretions. However, the ability of anesthesia providers to fully comply with these guidelines is questioned even by the authors who note that opportunities for hand hygiene can be more than 50 an hour, leading to failure of compliance up to 83% of the time.

**Figure 3:**
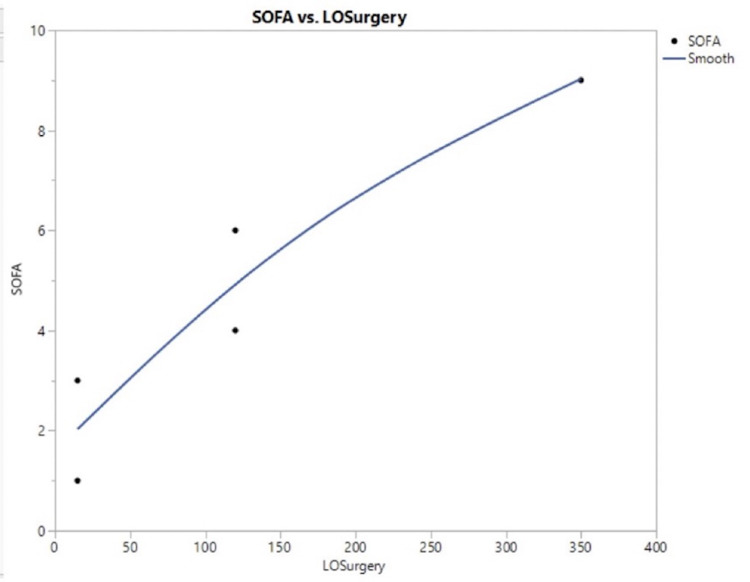
Severity of sepsis, as seen in the maximal SOFA score on the Y-axis, is correlated with the length of surgery. The correlation between SOFA score and length of surgery in minutes (length of surgery) on the x-axis; R = 0.933, P = 0.02. SOFA: Sequential Organ Failure Assessment; LOSurgery: length of surgery

An interesting observation in our series is the fact that the severity of the infection was correlated with the length of the procedure (Figure 3, Table 2). This may indicate that the duration of the patient’s exposure to the anesthesiologist, most likely his hands, was significant in increased bacterial burden and the development of the sepsis syndrome. Therefore, the transmission of infection was probably not due to a single point in time. Loftus et al. showed that contamination of the anesthesia workstation occurs in as little as four minutes after the start of anesthesia and that this contamination is closely related to contamination of stopcock hubs on the IV [23].

*Citrobacter freundii* is an uncommon pathogen of nosocomial infections, although it is frequently present in the environment. In hospitalized patients, *Citrobacter freundii* is the source of infection in only 0.7% of those with bacteremia [24]. It is also not a very frequent organism in the normal human microbiome. This fact is one of the strengths of this epidemiological report as it establishes the fact that the same organism was the cause of sepsis in all the patients, as well as the source was the anesthesiologist in whom the microbiome demonstrated the same organism. Because there was only one clinician involved in the care of all patients who was a carrier of the uncommon organism detected in the two patients with positive blood cultures establishes him as the only possible source of the infection.

Limitations

A major limitation of our epidemiological inquiry and assessment of the events is the fact that we have no clear explanation for the mechanism by which this infection occurred. Although breaches in the aseptic technique were identified in the physician’s work, we could not explain the occurrence of a cluster of patients over two days, without any change in practice just before the outbreak. We could not identify any special event regarding the anesthesiologist’s health in the days before the outbreak. Despite the fact that we have no mechanistic explanation for the infection of all patients, there is no doubt that the source of the infecting organism was the anesthesiologist.

The correlation between the severity of the infection and the duration of surgery, that is, the time when the anesthesiologist was caring for the patient invariably leads to the conclusion that the number of contacts between the anesthesiologist and the patient was a contributing factor to the development of sepsis. This indicates that the source of infection was through the intravenous line or through the airways. During anesthesia delivery, both airway management maneuvers and intravenous medication administration increase as the duration of the procedure increases. Therefore, we believe that the source of the infection was most likely intravenously, although we cannot rule out contact through the airways as a source.

Alverdy et al. recently reported that the fact that breaches of aseptic technique occur regularly in the operating room, yet infections following surgery are not that common, suggesting that the source of infection should be sought elsewhere. They propose an approach of sequencing organisms that were cultured from infected wounds and comparing them to the patient’s microbiome, which may lead to identifying the source of the infection in many cases and perhaps direct us toward different approaches to deal with surgical site infections [25].

## Conclusions

The complexity of infection control during anesthesia delivery is due to the intensity of the clinical activities, as well as the unique setup of the anesthesia workplace. When an infection occurs following surgery, its source may not always be apparent even when an epidemiological investigation is conducted. Others have reported on the role of anesthesia personnel in the transmission of pathogenic organisms from one patient to another due to lapses in sterile techniques. Our report establishes the role of the clinician’s microbiome as the source of infections in patients. This source of infection will usually be missed when utilizing conventional microbiological techniques when performing an epidemiological inquiry.

When there is an outbreak of infections following surgery, we propose that, in addition to exploring a patient as a source of infection, and performing standard cultures of the patients, environment, and the clinicians who were involved in patient care, an additional approach should be utilized. This involves the evaluation of the microbiome of the clinicians involved in the patient’s care. As in the outbreak described here, this may provide valuable information which can be significant in identifying the source of infections and decreasing nosocomial infections.
